# Resolving Transient Electron–Phonon Coupling with Time-Resolved Spontaneous Raman Spectroscopy

**DOI:** 10.1021/acs.jpclett.6c02648

**Published:** 2026-10-07

**Authors:** Guy Reuveni, Maya Levy Greenberg, Matan Menahem, Olle Hellman, Omer Yaffe

**Affiliations:** † Department of Chemical and Biological Physics, 34976 Weizmann Institute of Science, Rehovot 76100, Israel; ‡ Department of Molecular Chemistry and Material Science, 34976 Weizmann Institute of Science, Rehovot 76100, Israel

## Abstract

Understanding the interaction of charge carriers with lattice vibrations in the quasi-equilibrium regime is crucial for semiconductor functionality. However, the structural signatures of these interactions are often too subtle for conventional ultrafast techniques to detect. We developed a time-resolved spontaneous Raman technique based on time-correlated single-photon counting to track the spectral response following photoexcitation, providing subwavenumber spectral resolution and a subnanosecond temporal resolution. Unlike traditional pump–probe schemes, our method utilizes a modulated continuous-wave probe to maintain high spectral resolution, enabling detection of low-frequency Raman shifts down to 10 cm^–1^. Applied to lightly boron-doped silicon, we resolve *intra*-valence band and *inter*-valence band electronic transitions. A coupled-mode analysis of transient phonon asymmetry, resulting from interference with the intervalence band transitions, reveals electron–phonon coupling parameters that directly relate to carrier recombination. By capturing these subtle dynamical shifts, we demonstrate that this platform offers a powerful probe for investigating electron–phonon interactions in long-lived excited states.

## Graphical abstract

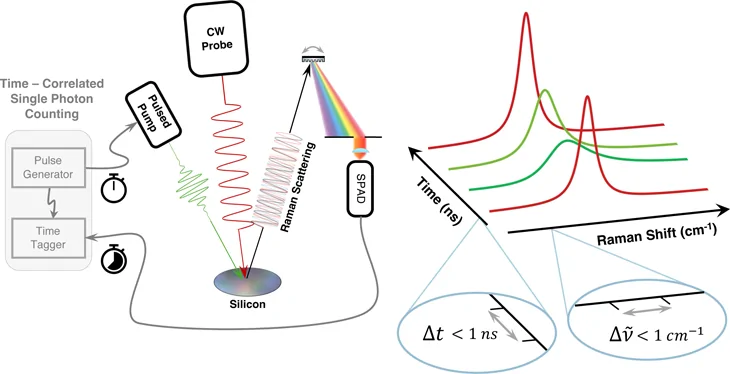

Following photoexcitation, hot carriers rapidly lose phase coherence and relax toward the band edges through carrier–carrier scattering and phonon emission on subpicosecond time scales. After this initial relaxation, electrons and holes accumulate near the bottom of the conduction band and the top of the valence band, forming a quasi-equilibrium carrier distribution. The carrier population then gradually decreases through recombination over time scales ranging from picoseconds to microseconds.^1,2^ This quasi-equilibrium stage is central to material functionality. During this period, processes such as transport of free carriers or polarons, self-trapping, and exciton dissociation occur through interactions between electronic and structural degrees of freedom.^3–6^

Common ultrafast spectroscopies predominantly track interactions between electronic and structural degrees of freedom by monitoring observables related to electronic properties. Transient absorption and time-resolved photoluminescence follow carrier densities and band-structure evolution,^7,8^ while terahertz spectroscopy probes carrier mobility and scattering dynamics.^9^ Coherent Raman techniques track impulsively driven vibrational modes, but their sensitivity diminishes once phase coherence is lost after a few picoseconds, limiting them largely to the hot-carrier cooling regime rather than the longer-lived quasi-equilibrium state.^10^

In contrast, time-resolved spontaneous Raman scattering directly probes vibrational and structural dynamics throughout this quasi-equilibrium regime.^11^ The temporal evolution of Raman frequency, line width, and intensity encodes changes in force constants, phonon lifetimes, and mode populations.^12,13^ Because the Raman cross section depends on the electronic polarizability, the technique is inherently sensitive to transient modifications of electron–phonon coupling and local bonding.^14^ It therefore provides direct access to how vibrational energy redistributes across the lattice and how electronic excitations reshape the structural landscape.^15,16^

The common approach to time-resolved spontaneous Raman scattering is a pump–probe scheme, in which an ultrafast pump pulse drives the system out of equilibrium and a time-delayed pulsed probe records the spontaneous Raman response.^11–13,15,17–28^ Clearly, the use of ultrashort pulses limits the spectral resolution of the Raman spectra. In principle, pump–probe Raman experiments can achieve high spectral resolution by using longer probe pulses with a narrow bandwidth. In practice, this is uncommon, and even when the probe pulse is long and spectrally narrow, amplified spontaneous emission and residual spectral wings near the laser line make efficient filtering difficult.^11,15^ These issues become especially severe when probing low-frequency Raman shifts below $∼100cm^{-1}$ , where strong suppression of stray light is required.

Here, we implement an alternative approach to time-resolved Raman scattering based on time-correlated single-photon counting (TCSPC), in which individual Raman photons are time-tagged relative to the pump excitation.^29–32^ By combining a modulated continuous-wave probe with TCSPC detection and a pulsed optical pump, we obtain a subnanosecond temporal resolution while maintaining high spectral resolution. We demonstrate this approach in silicon, where photoexcitation induces two Raman-active valence-band transitions. A low-frequency electronic Raman response, assigned to *intravalence-band* (intra-VB) scattering, appears at low shifts up to 200 cm^–1^,^33–35^ while *intervalence-band* (inter-VB) transitions overlap with the optical phonon at 521 cm^–1^, creating an asymmetric Fano line shape.^13,36–39^ High spectral resolution enables a coupled-mode analysis to extract electron–phonon coupling parameters, which track the photoexcited carrier density during recombination.

## TCSPC-Based Time-Resolved Raman Experimental Platform

Time-resolved spontaneous Raman scattering measurements are performed using a setup based on TCSPC detection, schematically shown in Figure 1(a). The instrument is built around a 1-m focal-length monochromator equipped with a 1800 grooves mm^–1^ grating. The spectrometer is coupled to an optical microscope operating in a backscattering configuration. A continuous-wave (CW) diode laser provides probe excitation at 785 nm. An acousto-optic modulator amplitude-modulates the probe beam at 20 kHz to generate an on–off sequence, enabling subtraction of pump-induced photoluminescence from the Raman signal. Nonequilibrium dynamics are initiated by a spatially coaligned pulsed optical pump centered at 515 nm. The pump delivers 64 ps pulses at a repetition rate of 10 MHz, with pulse energy of ∼0.9 nJ at the sample surface, corresponding to a fluence of approximately 30 mJ cm^–2^ for the ∼2-μm-diameter focal spot. Elastic scattering from the excitation line is suppressed using a beam splitter in combination with two volume holographic grating notch filters, enabling detection of low-frequency Raman shifts down to 10 cm^–1^.

**Figure 1:**
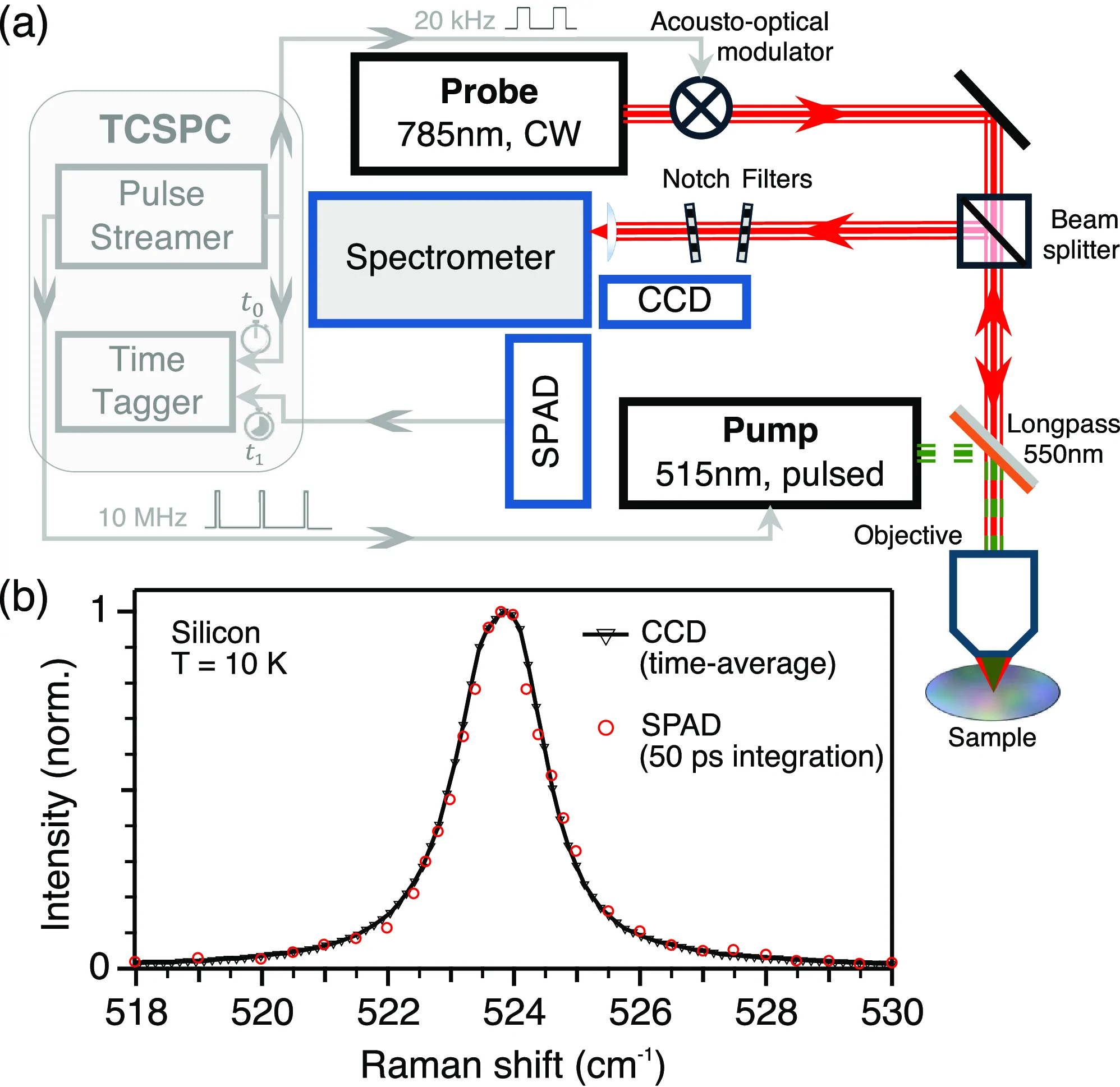
(a) Schematic of the time-resolved spontaneous Raman spectroscopy apparatus utilizing time-correlated single-photon counting. A modulated continuous-wave probe at 785 nm and a pulsed pump at 515 nm are coaligned onto the sample. (b) Normalized probe-only Raman spectra of the silicon optical phonon at 10 K are recorded using a CCD detector (black) and reconstructed from a single 50 ps time bin using the single-photon avalanche photodiode with TCSPC (red). The overlap demonstrates that introducing subnanosecond temporal resolution does not compromise spectral resolution.

The spectrally dispersed Raman signal is collected at the monochromator’s exit slit and detected by a single-photon avalanche photodiode (SPAD). The spectral resolution of the setup is determined by the slit’s variable width, allowing a predefined spectral bandpass to be collected on the SPAD. Here, it is set to ∼1.1 cm^–1^, but can be tuned as low as ∼0.14 cm^–1^ (see the Supporting Information). Photon arrival times are recorded with a time-tagging module with 50 ps time-binning, with synchronization between the laser source, modulation electronics, and detection system provided by a pulse generator. The overall temporal instrument response function, determined by the timing jitter of the spectrometer, detector, time tagger, and associated electronics, has a full width at half-maximum of ∼710 ps. While a transform-limited pulsed probe of comparable duration would, in principle, allow high spectral resolution in a conventional pump–probe scheme, generating such a narrowband probe from mode-locked ultrafast lasers requires additional pulse shaping. Alternatively, simpler Q-switched probes lack the required line width for low-frequency Raman measurements and introduce synchronization complexity that compromises temporal resolution.

A time-resolved Raman spectrum is obtained by sequentially scanning the monochromator grating. At each spectral position, TCSPC is used to accumulate a temporal histogram of Raman photon arrival times, from which the full time- and frequency-resolved Raman response is reconstructed. A detailed description of the experimental setup with the analysis method is found in the SI.

The temporal instrument response function does not represent a fundamental limitation of the method. Improved temporal resolution is expected with state-of-the-art low-jitter avalanche photodiode arrays, which would also enable operation in spectrograph mode, thereby significantly reducing the acquisition time for a full spectrum.^40,41^ Additional improvements are anticipated with advanced time-tagging electronics and shorter pump pulse durations. Figure 1(b) demonstrates that a spectrum reconstructed within a 50 ps temporal window retains the same spectral resolution as a time-averaged charge-coupled device (CCD) measurement. The silicon optical phonon is measured at 10 K under probe-only illumination, where its narrow line width provides a stringent test of spectral performance.

## Electronic and Phonon Dynamics in Photoexcited Silicon

To demonstrate the capabilities of high-resolution time-resolved spontaneous Raman spectroscopy, we study the dynamics of photoexcitation of lightly boron-doped silicon wafer (dopant concentration 1 × 10^14^ cm^–3^) with a native oxide layer as a model system.

Figure 2(a) displays the Stokes and anti-Stokes transient Raman response for an estimated probe-volume-averaged excess carrier density of approximately 7 × 10^18^ cm^–3^, averaged over the first 0.5 ns after excitation at 280 K (refer to the SI for similar data across a temperature range of 10–280 K). In the top panel, the spectrum before excitation (shown in red and averaged over the interval from −0.5 to 0 ns) is compared with the spectrum averaged over the period from 2.35 ns to 2.85 ns after excitation (shown in green). The bottom panel presents the differential spectra Δ*I*(ω, *t*) = *I*(ω, *t*) – *I*_*∞*_, where *I*_*∞*_ refers to the spectrum prior to excitation. The most significant distortion occurs at *t* = 2.35 ns, revealing two correlated features: a broad low-frequency Raman scattering signal that extends to approximately 200 cm^–1^, and a marked modification of the zone-center optical phonon at 521 cm^–1^.

**Figure 2:**
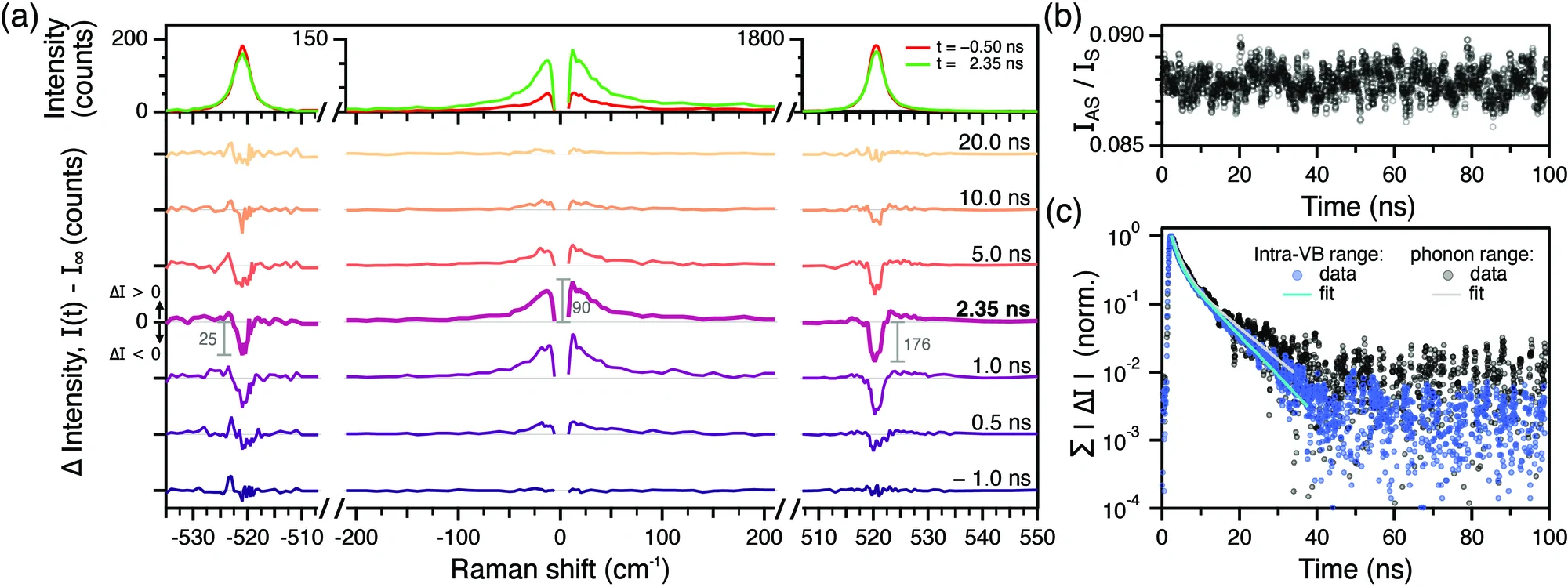
Time-resolved spontaneous Raman response of lightly boron-doped silicon at 280 K. (a) Top panel: Raman spectrum of low-doped silicon pre-excitation (red) and post-excitation (green). Bottom panel: Differential spectra Δ*I*(ω, *t*) obtained by subtracting the pre-excitation spectrum from each transient spectrum. The bold spectrum at *t* ≈ 2.35 ns represents the maximum distortion. Differential spectra are offset for clarity, with gray axes indicating the zero level and gray scale bars representing the Δ*I* magnitude for each spectral range. The data reveal a transient amplification of the low-frequency *intravalence-band* (intra-VB) signal and a time-dependent modification of the optical phonon near 521 cm^–1^ due to the *intervalence-band* (inter-VB) transitions. (b) Time-dependent anti-Stokes/Stokes amplitude ratio. The constant ratio verifies that no significant lattice heating occurs during the measurement. (c) Temporal evolution of the normalized integrated absolute differential intensity *∑*_ω_|Δ*I*|, integrated over the intra-VB (10–200 cm^–1^, blue) and phonon (510–560 cm^–1^, black) spectral ranges, shown with biexponential fits.

The broad low-frequency response is assigned to photoinduced free-carrier intraband electronic Raman scattering. Its spectral form and range are consistent with the intra-VB continuum occurring at low Raman shifts up to approximately 200 cm^–1^, previously observed in *p*-Si and attributed to free carriers in the upper valence bands.^33–35^ Concurrently, a continuum of inter-VB hole transitions begins near 355 cm^–1^ and extends to higher energies, overlapping with the optical phonon at 521 cm^–1^.^13,36–39,42^ The interference between these transitions and the discrete phonon transition produces the characteristic asymmetric transient Fano line shape.

Figure 2(b) demonstrates that these spectral changes are not caused by lattice heating. The anti-Stokes/Stokes amplitude ratio, which provides direct lattice thermometry,^11,25,43,44^ remains constant within experimental uncertainty, indicating that the lattice temperature does not increase during the transient response. Figure 2(c) shows the temporal evolution of the normalized, integrated absolute differential intensity, *∑*_ω_|Δ*I*(ω, *t*)|, evaluated over the intra-VB Raman range (10–200 cm^–1^, blue) and the phonon range (510–560 cm^–1^, black). The latter is assigned to the optical phonon, the inter-VB transitions, and their mutual interference. Both signals rise promptly after photoexcitation and decay toward equilibrium, tracking the transient carrier population via a biexponential decay typical of carrier recombination in silicon.^45–47^ Focusing on the dominant fast component, we extract decay constants of τ_1_ = 1.8 ns for the intra-VB signal and τ_1_ = 2.7 ns for the phonon feature. While these values differ, they are of the same order of magnitude and are consistent with effective carrier lifetimes in lightly doped silicon with a native oxide, where surface recombination dominates.^48^

## Transient Electron–Phonon Interactions

Next, we turn to the additional information encoded in the transient Raman line shape, which becomes accessible because of the high spectral resolution of the present measurements. We focus on the optical phonon and the associated inter-VB region, as the low-frequency intra-VB electronic response is spectrally broad and featureless, thus precluding a meaningful decomposition into distinct components.

Although the asymmetric phonon peak in the presence of photoexcited carriers is often described within the Fano formalism, we do not adopt that approach here. In the conventional Fano model, the discrete excitation is treated as a stationary eigenstate whose spectral width arises solely from its interaction with a continuum of electronic transitions.^49^ Within this framework, the phonon line width is rigidly tied to the strength of the coupling to the inter-VB transitions and does not account for the intrinsic phonon lifetime, which exists independently of the electronic continuum and exhibits a pronounced temperature dependence (see the Supporting Information). Moreover, the Fano formalism is constructed to diagonalize the energy matrix of stationary states and does not natively describe the time-dependent evolution of a coupled system. In our measurements, the phonon line shape evolves continuously from an asymmetric profile shortly after excitation to a nearly symmetric form at longer delay times as the carrier population decays. This transient relaxation of the spectral asymmetry cannot be captured by a model based solely on static energy eigenvalues.

Instead, we employ a general description based on two coupled Lorentzian modes,^50^ consisting of a narrow mode representing the optical phonon and a broad mode representing the inter-VB transitions. This approach separates intrinsic phonon lifetime effects from coupling-induced distortions and provides a unified framework for analyzing the temporal dependence of the spectra. Formally, the system is described by a Green’s function *G*(Ω),^51^ as previously implemented for coupled-mode systems.^52–54^ The Raman intensity is proportional to the corresponding spectral function, given by the imaginary part of the Green’s function weighted by the Raman polarizability derivatives,

1 $$I\left(\omega ,T\right)\propto n\left(\omega ,T\right)\underset{ij}{\sum }\left[Im\left\{G_{ij}\left(\omega ,T\right)\right\}\chi_{i}\chi_{j}\right]$$

where χ_1_ and χ_2_ denote the polarizability derivatives associated with the phonon and the inter-VB transitions, respectively. The interacting Green’s function follows from the Dyson equation,

2 $$G{\left(\Omega \right)}^{-1}=g^{0}{\left(\Omega \right)}^{-1}+\Sigma \left(\Omega \right)$$

where *g* ^0^(Ω) is the harmonic Green’s function and Σ­(Ω) is the self-energy. For two coupled modes with frequency-independent parameters,^52,53^ these matrices take the form

3 $$g^{0}{\left(\Omega \right)}^{-1}=\left(\begin{array}{ll} \omega_{1}^{2}-\Omega^{2} & 0\\ 0 & \omega_{2}^{2}-\Omega^{2} \end{array}\right)$$

4 $$\Sigma \left(\Omega ,t\right)=\Omega \left(\begin{array}{ll} -i\Gamma_{1} & \delta \left(t\right)+i\gamma \left(t\right)\\ \delta \left(t\right)+i\gamma \left(t\right) & -i\Gamma_{2} \end{array}\right)$$

Here, Ω is the probing frequency, ω_1_ and ω_2_ are the bare frequencies of the phonon and inter-VB transitions mode, Γ_1_ and Γ_2_ are their respective damping constants, and δ­(*t*) and γ­(*t*) respectively represent the real and imaginary parts of the time-dependent intermode coupling.

To ensure physical robustness and avoid overparameterization, we impose several constraints in the fitting procedure. The central frequency and width of the inter-VB transitions are fixed at ω_2_ = 700 cm^–1^ and Γ_2_ = 400 cm^–1^, reflecting the broad inter-VB Raman response of silicon that overlaps the optical phonon near 521 cm^–1^.^37,42^ The relatively modest phonon spectral distortion induced by photoexcitation supports keeping these parameters constant throughout the transient. The inter-VB transitions scattering strength χ_2_ is fixed at 20, as the fits are not sensitive to its precise value. The phonon frequency ω_1_, damping Γ_1_, and scattering strength χ_1_ are fixed to the Lorentzian parameters extracted from the spectrum prior to the excitation. This procedure assumes that the transient modifications arise from coupling to the inter-VB transitions rather than from changes in the intrinsic phonon lifetime.

Under these constraints, the only free parameters are the coupling terms δ­(*t*) and γ­(*t*). Physically, both are expected to scale with the photoexcited carrier concentration, since the effective electron–phonon interaction strength is proportional to the density of free carriers. We therefore treat them as time-dependent quantities that implicitly incorporate the carrier population. The real part δ couples the mode frequencies, while the imaginary part γ couples their damping channels and represents a dissipative interaction.^55^

The results of this analysis are summarized in Figure 3. Figure 3(a) shows the coupled-modes fit to the excited-state spectrum, 2.35 ns after excitation, when the photoexcitation effect peaks. The cross term between the phonon and inter-VB transitions, shown in red, dominates the asymmetry, whereas the direct contribution of the inter-VB transitions (green) is negligible. Figure 3(b) presents the statistical *R* ^2^ of the coupled-mode fit, compared with that of a simple Lorentz oscillator model. The superior performance of the coupled-mode description highlights the transient contribution of the coupling to the inter-VB transitions and its temporal decay, demonstrating that the coupled-mode model is required to reproduce the asymmetric phonon profile. Figure 3(c) shows the temporal evolution of δ­(*t*) and γ­(*t*). Dashed curves in Figure 3(b) and 3(c) are the biexponential function fits to the transients.

**Figure 3:**
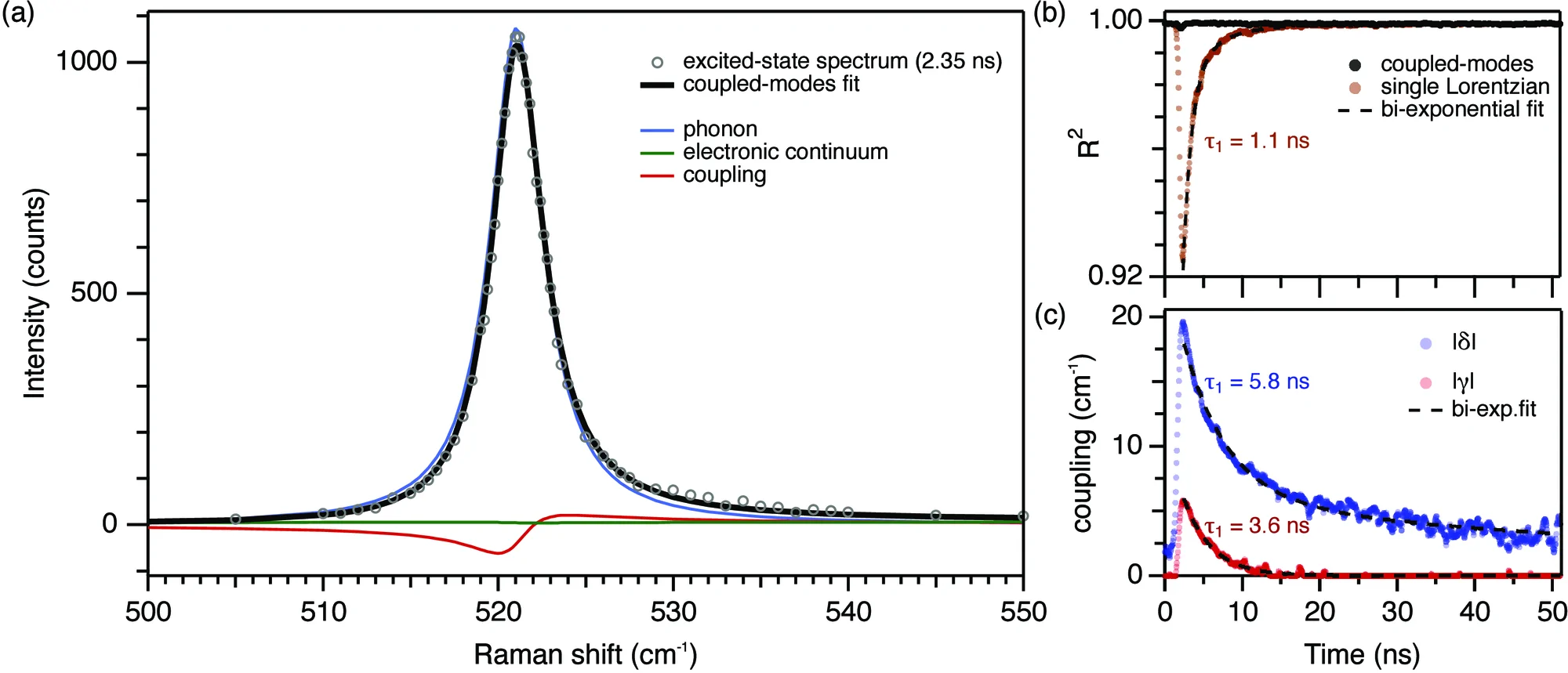
Time-resolved line shape analysis of the optical phonon and the *inter*-valence-band (inter-VB) spectral region in lightly boron-doped silicon at 280 K. (a) Raman spectrum 2.35 ns after photoexcitation. Gray circles indicate the time-averaged spectrum integrated over 2.35–2.85 ns, shown alongside the two-coupled-modes fit (black). The asymmetry of the peak is apparent compared to the purely phononic contribution (blue), with most of the asymmetry originating from the cross terms (red) between the inter-VB transitions and the phonon. The direct contribution from the inter-VB electronic transitions (green) is negligible. (b) Statistical *R* ^2^ for the coupled-mode fit (black), compared with a single Lorentz oscillator fit (brown). (c) Temporal evolution of the absolute real (|δ|, blue) and imaginary (|γ|, red) coupling parameters. The dashed curves in panels (b) and (c) represent biexponential fits to the transients.

The extracted decay constants (τ_1_) for *R* ^2^(*t*), δ­(*t*), and γ­(*t*) are not identical. This deviation from a single scaling factor reflects the fact that δ and γ enter the Raman intensity expression in eq 1 with different functional dependence, and the statistical measure *R* ^2^ is not expected to vary linearly with carrier concentration. Nevertheless, the overall agreement in their temporal behavior supports the interpretation that the transient line shape evolution is governed by the decay of the photoexcited carrier population.

In summary, high-resolution time-resolved spontaneous Raman spectroscopy reveals the dynamics of electron–phonon interactions in photoexcited silicon during the quasi-equilibrium regime. A coupled-mode analysis shows that the transient asymmetry of the optical phonon arises from its time-dependent interaction with the inter-VB transitions. The coupling parameters δ­(*t*) and γ­(*t*) quantify how the photoexcited carrier population modifies the phonon properties, and their decay directly follows the reduction of the carrier density during recombination.

Our work addresses the challenge of resolving the subtle interactions between charge carriers and lattice vibrations during the quasi-equilibrium regime of semiconductors. By implementing a time-correlated single-photon counting (TCSPC) approach with a modulated continuous-wave probe, we achieve subwavenumber spectral resolution and a subnanosecond temporal resolution. This allows for the detection of low-frequency shifts and high-frequency phonon modifications. We demonstrated the effectiveness of this technique in lightly boron-doped silicon, resolving the transient amplification of the *intra*-valence-band transitions and the time-dependent interference between the optical phonon and the *inter*-valence-band transitions. Using a coupled-mode analysis, we extracted time-dependent coupling parameters, δ­(*t*) and γ­(*t*), which directly track the carrier recombination dynamics. This experimental platform offers a versatile high-resolution probe for investigating the complex electron–phonon interactions that define the functional properties of modern semiconductors.

## Supplementary Material



## Notes

Additional experimental details, supporting calculations, and extended data analysis procedures, including temperature-dependent measurements and line shape modeling (PDF)
