# Supplementary material for: Resolving Transient Electron–Phonon Coupling with Time-Resolved Spontaneous Raman Spectroscopy
Source: J Phys Chem Lett. 2026 Oct 7;17(42):12132–8. doi: 10.1021/acs.jpclett.6c02648 (PMC13700098; doi:10.1021/acs.jpclett.6c02648)
Supplement: Supplementary file 1 [file jz6c02648_si_001.pdf]

Supplementary Information

Resolving Transient Electron–Phonon Coupling with Time-Resolved Spontaneous Raman Spectroscopy

Guy Reuveni,[1] Maya Levy Greenberg,[1] Matan Menahem,[1] Olle Hellman,[2,*] and Omer Yaffe[1,†]

[1]*Department of Chemical and Biological Physics, Weizmann Institute of Science, Rehovot 76100, Israel;*

[2]*Department of Molecular Chemistry and Material Science, Weizmann Institute of Science, Rehovot 76100, Israel;*

(Dated: September 19, 2026)

* olle.hellman@weizmann.ac.il

† omer.yaffe@weizmann.ac.il

S1. EXPERIMENTAL

S1.a. TCSPC Time-Resolved Raman Experimental Platform

The time-resolved Raman scattering measurements were carried out using a setup developed by extending our existing high-resolution Raman platform[1,2] (Figure 1 in the main article). The system is built around a 1 m focal-length monochromator (FHR 1000, Horiba), with a 1800 grooves mm^{-1} grating. The Raman probe laser is a 785 nm continuous-wave diode laser (XTRA II, Toptica Inc., USA), which is spectrally cleaned with amplified spontaneous emission filters and subsequently spatially filtered. The probe beam is directed into an acousto-optical modulator (M1206-P110L-1, ISOMET), where the first order, amplitude-modulated diffracted beam is taken downstream towards the sample, producing an on/off sequence at 20 kHz. The 515 nm pulsed pump laser (LDH-P-FA-515L, PicoQuant) operates at a repetition rate of 10 MHz with a pulse duration of approximately 64 ps, and passes through a telescope to ensure that the pump and probe focal points are precisely overlapping at the sample surface. The average pump power measured immediately before the microscope objective during the experiments was 10-13 mW. The transmission of the microscope objective at 515 nm is 83%, giving an estimated incident power at the sample of 8.3-10.8 mW, corresponding to 0.83-1.08 nJ per pulse. The pump focal-spot diameter was estimated to be ~ 2 μm, and the area-normalized incident fluence at the sample surface is approximately 26-34 mJ cm. The pump beam is merged with the central optical path via a dichroic mirror (DMLP550R, Thorlabs), spatially co-aligned with the probe beam, and focused onto the same spot on the sample. Both pump and probe lines are focused on the sample through an optical microscope with 0.55NA/50x objective (LD EC Epiplan-Neofluar 50x/0.55 DIC M27, Zeiss) in a back-scattered configuration, passing through a half-wave plate (Thorlabs) to align the excitation polarization with the crystal axis to maximize Raman intensity. The sample is measured in a cryogenic-cooled cryostat (Janic Inc.) under high vacuum. Elastic scattering from the excitation line is suppressed using two volume holographic grating notch filters with OD>4 (SureBlock, Coherent) in combination with a beam splitter (NoiseBlock, Coherent), enabling reliable detection of Raman shifts down to 10 cm^{-1} from the laser line.

The spectrally dispersed Raman signal is collected at the width-variable entrance slit of the monochromator and detected using either a CCD (Synapse+, Horiba) or a single-

photon avalanche photodetector (Excelitas, SPCM-AQRH-46) with an additional exit slit that filters the bandwidth that enters it. Slit widths were set to 300 μm for the entrance slit and varied between 200-300 μm for the exit slit in the experiments in the main manuscript. Exit slit width values were chosen to balance between sufficient spectral resolution for the acquired spectrum and the total acquisition time.

Photon arrival times to the SPAD are recorded with a time-tagging module (Ultra 4 Value, Swabian Instruments), with synchronization between the laser sources, modulation electronics, and detection system provided by a Pulse Streamer 8/2 (Swabian Instruments). The time tags are recorded in 50 ps time bins, and the overall instrument temporal response function has a full-width at half-maximum of 710 ps, derived from the timing jitter of the pump laser, the electronics, the SPAD, and the diffraction grating (see fig. S1).

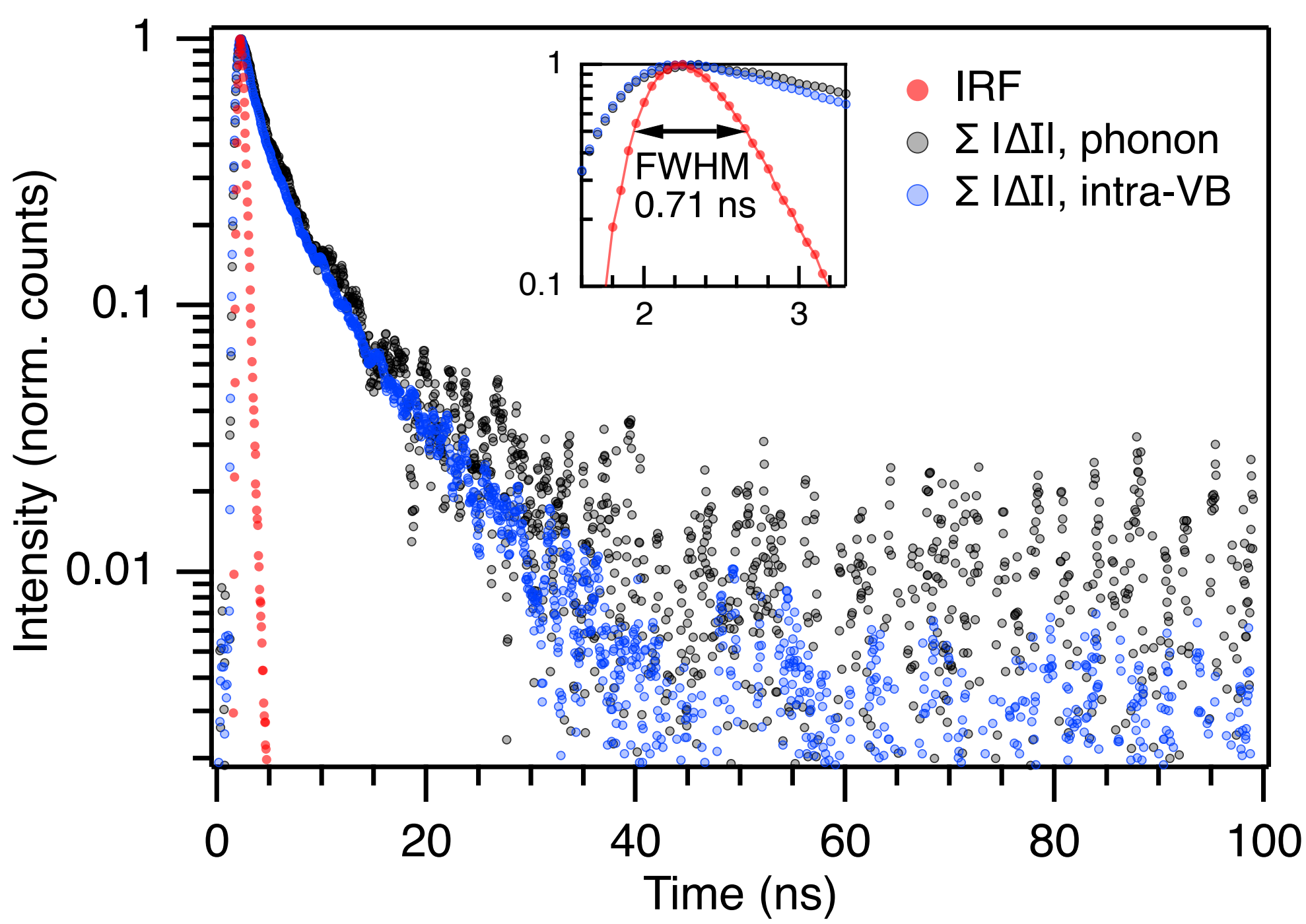

FIG. S1: The time-resolved Raman platform temporal instrument response function (IRF) is plotted (red markers), alongside the normalized integrated absolute differential intensity curves ($\sum_\omega |\Delta I|$), as shown in the main manuscript, of the intra-VB (10–200 cm^{-1}, blue) and phonon (510–560 cm^{-1}, black) spectral ranges. Inset - zoomed-in view around the FWHM of the IRF curve, showing a FWHM of 0.71 ns.

A complete time-resolved Raman spectrum is obtained by sequentially scanning the monochromator grating. At each spectral position, time-correlated single-photon counting is used to accumulate a temporal histogram of Raman photon arrival times for 20 minutes, from which the full time- and frequency-resolved Raman response is reconstructed. Average

laser power used for all measurements varied between 12-30 mW for the probe laser and 10-13 mW for the pump laser.

S1.b. Spectral Resolution

The effective spectral resolution of the setup is determined by the spectrometer's linear dispersion and the widths of the entrance and exit slits. The system operates as a monochromator, utilizing the 1800 gr/mm grating which provides a linear spectral dispersion (R_d) of 0.35 nm/mm around 785 nm.[3]

Because the signal is filtered through a variable exit slit and coupled to a single-pixel SPAD, the spectral resolution is strictly defined by the mechanical bandpass rather than spatial detector sampling. For a typical source image width of 50 μm at the entrance slit with an excitation wavelength of 785 nm and a typical exit slit width of 200 μm, the spectral resolution $\Delta\nu$ in wavenumbers is given by:

$$\Delta\tilde{\nu} = \frac{\Delta\lambda}{\lambda^2} = \frac{200\ (\mu m) \cdot 0.00035(\frac{nm}{\mu m}) \cdot 10^7(\frac{nm}{cm})}{(785\ nm)^2} \simeq 1.1cm^{-1} \tag{S1}$$

This is merely the estimated spectral resolution in the experiments presented in the main manuscript; higher spectral resolution can be achieved by decreasing the slit width even further, albeit at the expense of signal throughput. For comparison, in the case of a highly resolved spectrum at cryogenic temperatures, one can set an exit slit width of 25 μm to achieve $\Delta\tilde{\nu} \simeq 0.14cm^{-1}$.

S1.c. Silicon sample

The Silicon sample is a $< 100 >$ cut, Boron-doped with resistivity 70-85 Ω cm which translates into dopant concentration of $\sim 1 \cdot 10^{14}$ atoms cm^{-3} (Virginia Semiconductors). The Silicon wafer has a native oxide layer.

S2. CALCULATION OF THE TIME-DEPENDENT CARRIER DENSITY

The number of photoexcited carriers was estimated from the experimentally measured pump power. The average pump power immediately before the microscope objective was

10-13 mW. Accounting for the objective transmission at 515 nm ($T_{\mathrm{obj}} \simeq 0.83$), the estimated pulse energy incident on the sample at a repetition rate of 10 MHz is $E_{\mathrm{pump}} \simeq 0.83$-$1.08$ nJ.

The optical constants of silicon at 515 nm were taken from Ref.[5]. Using $\alpha_{515} = 9.25 \times 10^3$ cm^{-1} and the corresponding complex refractive index gives a surface reflectivity of approximately $R \simeq 0.38$. Assuming that each absorbed photon generates one electron–hole pair, the number of initially generated holes per pulse is estimated as

$$N_0 = \frac{(1-R)E_{\mathrm{pump}}}{hc/\lambda_{\mathrm{pump}}}, \tag{S2}$$

yielding $N_0 \simeq (1.3\text{–}1.7) \times 10^9$. We use $N_0 = 1.5 \times 10^9$ as the representative value in the calculation.

The carriers are initially generated within the approximately $1/\alpha_{515} \simeq 1.08$ μm absorption depth of the 515-nm pump. For the estimated 1 μm pump radius, the characteristic initial density is therefore of order 10^{20} cm^{-3}. This local initial density should be distinguished from the substantially lower carrier density sampled by the larger Raman probe volume after diffusion.

To model the spatial evolution of the carrier distribution, we use a three-dimensional Green's-function solution including both diffusion and recombination,

$$n(r,z,t) = \frac{N_0}{(4\pi D t)^{3/2}} \exp\left[-\frac{r^2 + (z-z_0)^2}{4Dt}\right] \exp\left(-\frac{t}{\tau}\right), \tag{S2}$$

where $D = 12$ cm^2 s^{-1} is the hole diffusion coefficient[4], r and z are the radial and axial coordinates, and $z_0 = 0.5\alpha_{515}^{-1} \simeq 0.54$ μm represents the center of the initially excited region. A recombination lifetime of $\tau = 1.5$ ns is used, consistent with the dominant nanosecond decay observed experimentally.

The observable carrier density is calculated by averaging $n(r,z,t)$ over the effective Raman probe volume. The probe depth is approximated by the 785-nm absorption depth, $h_{\mathrm{probe}} = \alpha_{785}^{-1}$, with $\alpha_{785} = 9.65 \times 10^2$ cm^{-1} [5]. The probe volume is modeled as a truncated cone determined by the approximately 1 μm beam radius at the surface and the collection angle inside silicon, $\theta = \sin^{-1}(\mathrm{NA}/n_{\mathrm{Si}})$, with NA $= 0.55$ and $n_{\mathrm{Si}} = 3.6$.

The instantaneous probe-volume-averaged carrier density is

$$\langle n(t) \rangle = \frac{1}{V_{\text{probe}}} \iiint_{V_{\text{probe}}} n(r, z, t)\, dV. \tag{S3}$$

Because the experimental spectrum referred to in the main text is averaged over the first 0.5 ns following excitation, we additionally calculate

$$\overline{n}_{0-0.5\,\text{ns}} = \frac{1}{0.5\text{ ns}} \int_0^{0.5\,\text{ns}} \langle n(t) \rangle\, dt. \tag{S4}$$

For the experimentally estimated range of N_0, the resulting value is approximately (6.0–7.8) $\times$ 10^{18} cm^{-3}. We therefore quote an effective carrier density of approximately 7×10^{18} cm^{-3} in the main manuscript. This value is a spatial and temporal average over the Raman sampling volume and the first 0.5 ns after excitation; it should not be interpreted as the initially generated local carrier density.

S3. DATA ANALYSIS

S3.a. Preliminary data treatment - background photoluminescence and noise subtraction

The raw data consist of two time-resolved Raman-spectra matrices, corresponding to the on/off modulated probe signal: one acquired with both pump and probe beams (fig. S2(a)), and one with the pump only (fig. S2(b)). Subtracting the pump-only matrix from the pump+probe matrix removes photoluminescence and other pump-induced backgrounds, as shown in the spectra at the top of the two matrices, which represent a spectrum at a specific time bin of 2.00-2.05 ns. The resulting difference matrix (fig. S2(c)) isolates the Raman background, along with any additional modifications of that background not directly attributable to the pump excitation, such as electron-phonon interaction effects on the phonon lineshape. These modifications in the difference matrix are not visible to the naked eye, here specifically because they are very subtle. To that end, we define a time-smoothing window of 10 spectra, each assigned to 50 ps, and, in total, equivalent to 500 ps, and apply a moving average throughout the time-dependent data. The time-averaged difference matrix serves as the starting point for all subsequent analyses. We extract the spectrum in the last time bin of the averaged difference matrix, referred to as the pre-excitation spectrum, and sub-

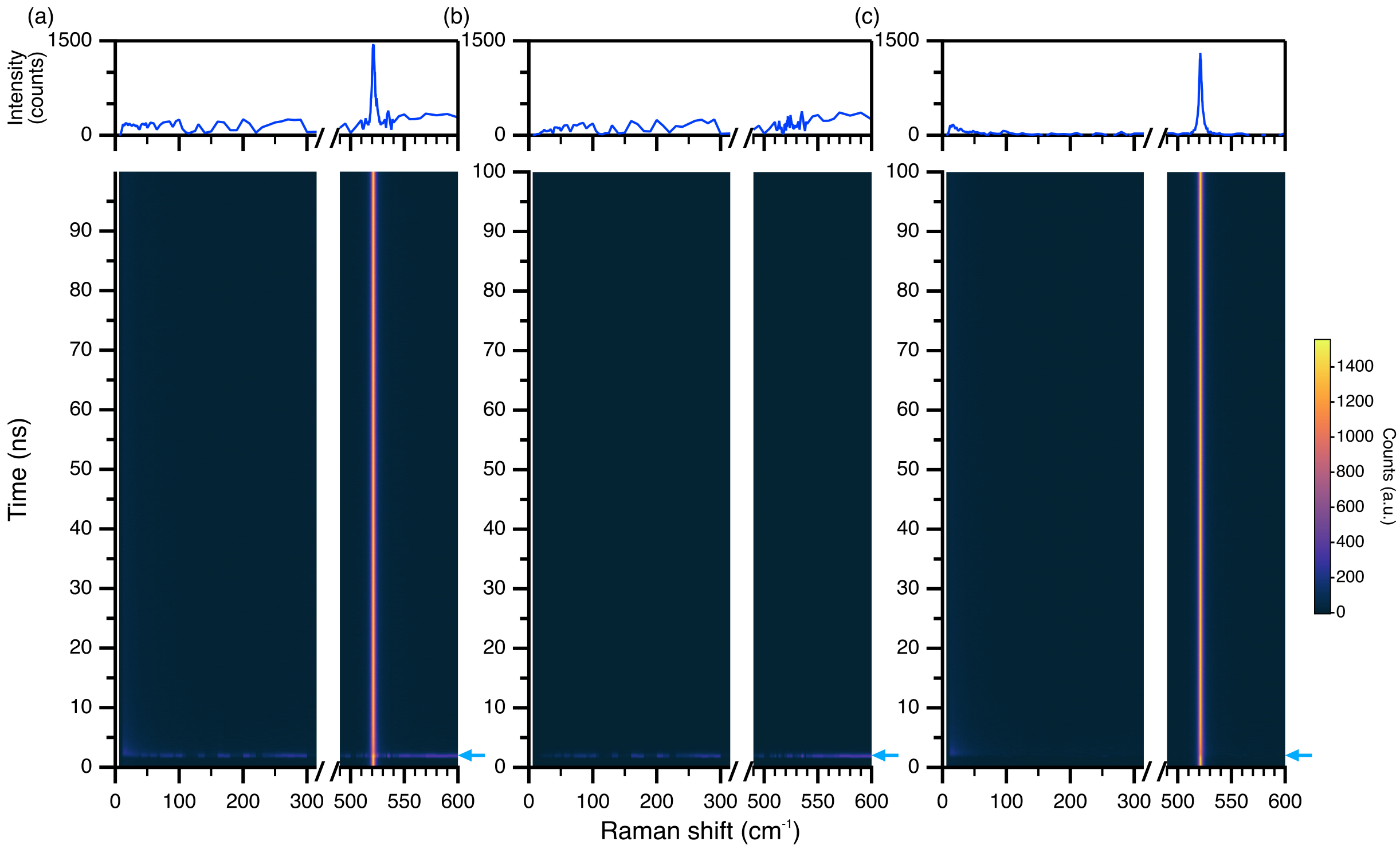

FIG. S2: Time-resolved Raman measurement scheme demonstrates the efficiency of the photoluminescence and background subtraction. Raw data at 280 K are presented as false-color heatmaps, with brighter colors indicating higher intensity. The time-resolved Raman data is collected under (a) simultaneous illumination of the pump and probe lasers and (b) under pump laser only, when the probe laser amplitude was off-modulated at the AOM. (c) The subtraction of the pump-only matrix from the pump+probe matrix yields the time-resolved Raman matrix that is used as the starting point for all subsequent analyses in this paper. Top panels - single Raman spectra reconstructed at the 2.00 ns time bin, marked with blue arrows on the heatmaps.

tract it from all other time-dependent averaged spectra to get the differential spectra matrix (fig. S3).

S3.b. temperature-dependent integrated differential intensity

To quantify and compare the dynamics of the photoexcited effect on the two electronic transitions and the effect on the optical phonon in the studied temperature range, each differential intensity spectrum $\Delta I(\omega, t)$ was integrated over the two spectral regions associated with the carrier-induced response, namely the low-frequency *intra*-valence-band (intra-VB) range (10–200 cm^{-1}) and the optical phonon range (510–560 cm^{-1}) that is modified by interference with the *inter*-valence-band (inter-VB) transitions. The full time-resolved, background-subtracted differential Raman spectra matrix at 280 K is shown in fig. S3 as a

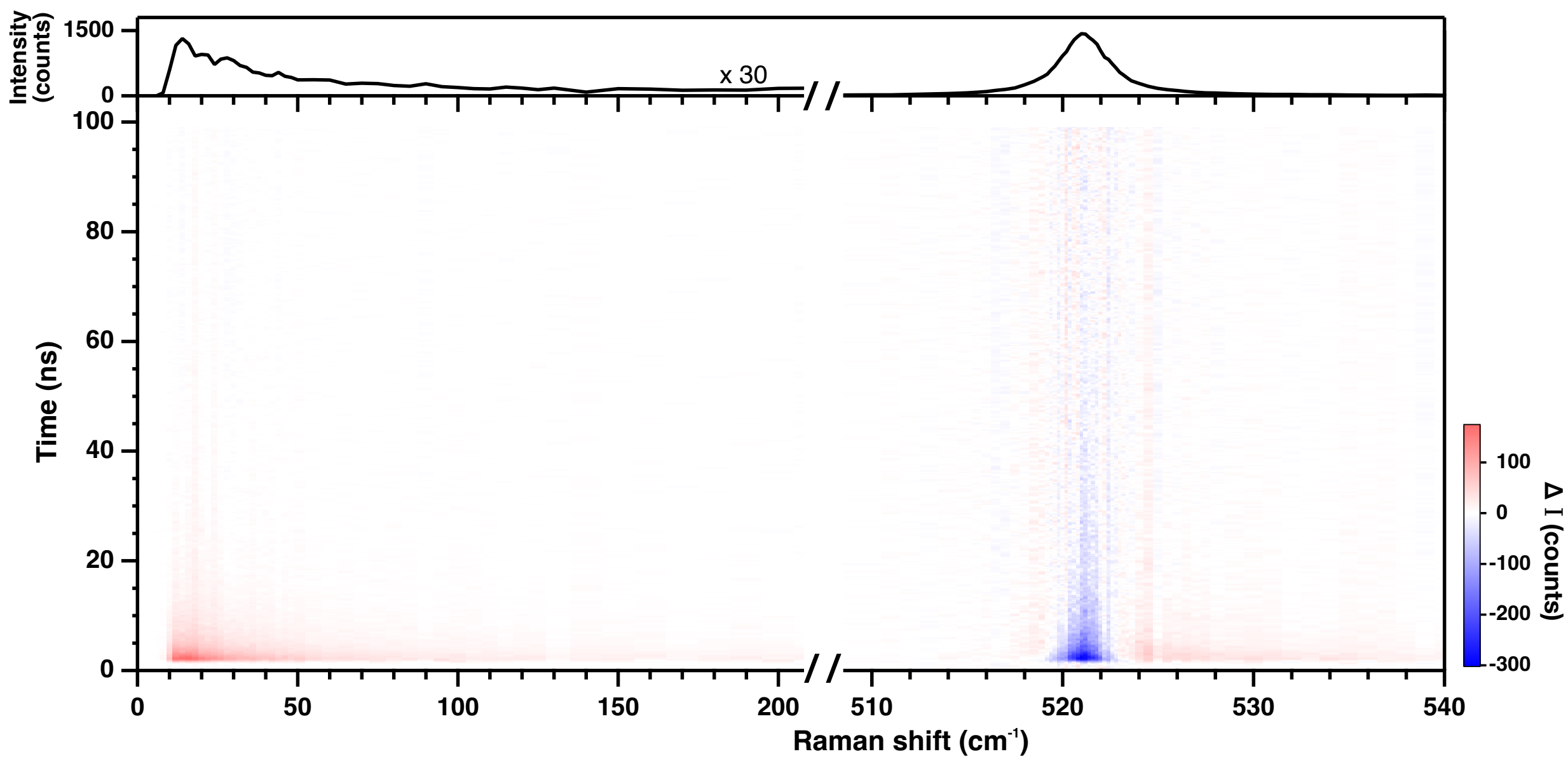

FIG. S3: Time-resolved spontaneous Raman response of lightly boron-doped silicon. Heatmap of the Stokes differential Raman intensity $\Delta I(\omega, t)$ at 280 K, defined relative to the pre-excitation spectrum (top), showing a transient low-frequency electronic continuum and a time-dependent modification of the optical phonon near 521 cm^{-1}.

false-color heatmap, where red and blue indicate signal enhancement or suppression, respectively, compared to the pre-excitation spectrum, shown in the top panel. This figure shows an example of the data used to extract the transient absolute integrated intensity. It shows a prompt response right after excitation ($t = 0$ ns), followed by an exponential decay that almost vanishes within 30 ns, returning to the pre-excitation spectrum. The low-frequency electronic Raman signal is amplified by the elevated density of excited charge carriers, which scatter light inelastically. The zone-center phonon around 521 cm^{-1} is distorted by coupling to the excited inter-VB transitions, as explained in the main article, showing an intensity quench at the peak (dark blue) and an increasing asymmetric signal on its higher-frequency side (light red).

The integrated differential intensity transient at each temperature was background-subtracted to eliminate different noise levels between temperatures, normalized, and fitted to a bi-exponential decay function, as shown in fig. S4 in gray curves; its results are annotated next to each decaying curve. The transients were fitted in the time range between the peak value and the first reaching the pre-excitation value. The resulting temperature-dependent decay constants τ_1 and τ_2 are presented in the bottom-right corner of each subfigure. At lower temperatures (≤ 30 K), the slow component is negligible, and only one decay constant

is effectively dominant.

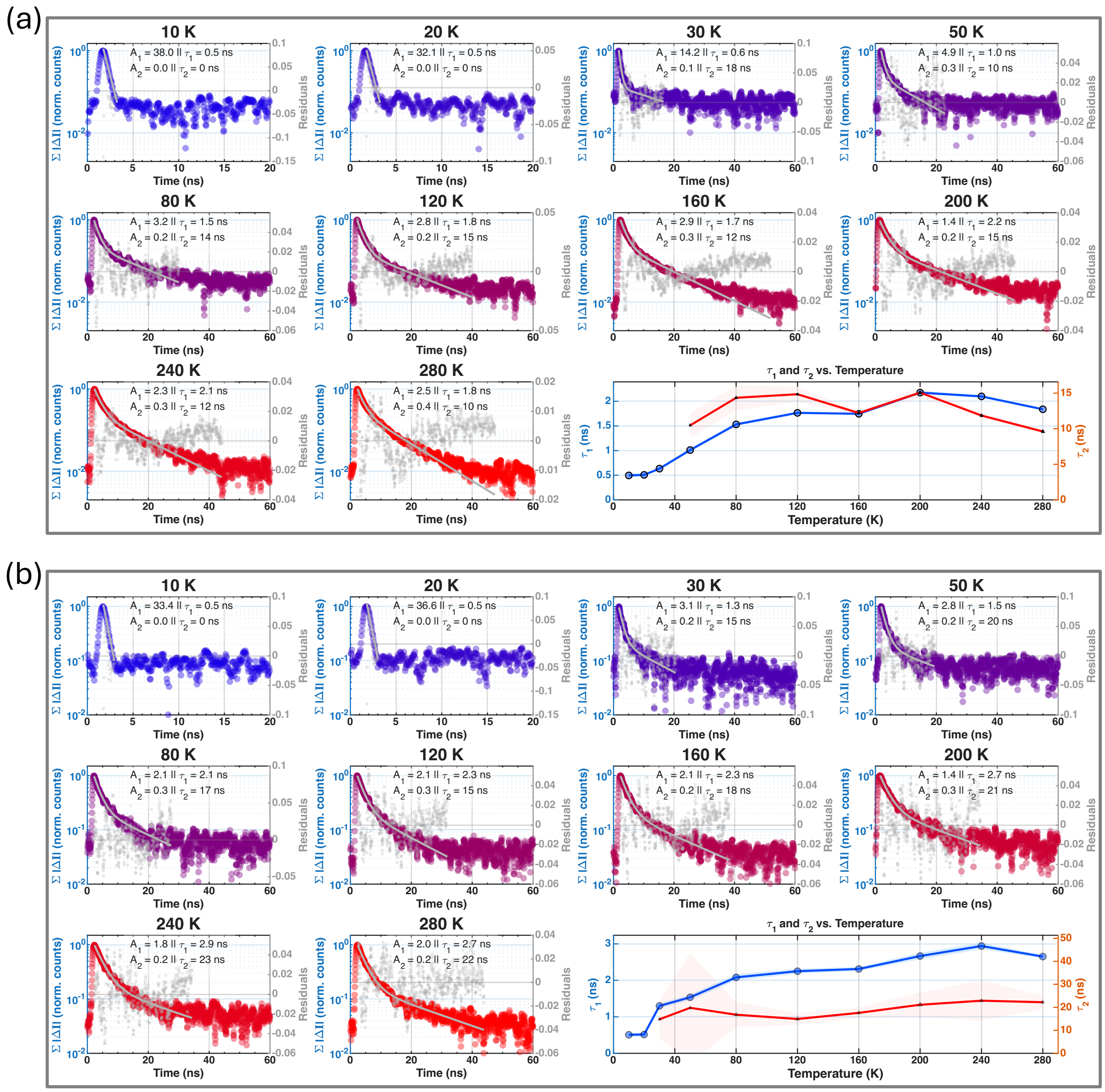

FIG. S4: Temperature-dependent integrated absolute differential intensity $\sum_\omega |\Delta I(\omega, t)|$ of (a) the intra-VB range and (b) the phonon range. Gray curves are the bi-exponential decay fit per transient, and gray circles are the fit residuals. The amplitude and decay constants are annotated next to the transients, and summarized at the bottom-right corners with the fast and slow decay constants plotted in blue and red, respectively. Shading represents the fit error of the decay constants, and the relative marker size represents the ratio of the fast and slow exponential amplitudes at each temperature.

S3.c. Lineshape analysis

1. Fano resonance model

The Fano interference model describes a system in which a discrete scattering state, the optical phonon, interacts through energy exchange with a continuum background of electronic transitions, here, the inter-valence band hole transitions[6,7]. The temperature- and time-dependent data were fitted to a Fano lineshape of the form:

$$I = I_0 \frac{(q+\varepsilon)^2}{1+\varepsilon^2} \tag{S3a}$$

$$\varepsilon = \frac{\omega - \omega_0}{\frac{\Gamma}{2}} \tag{S3b}$$

where I_0 is an intensity prefactor, q the Fano asymmetry parameter, ω the measured frequency, ω_0 the resonance frequency, and Γ the squared matrix element of the coupling between the continuum and the discrete states[6,8], experimentally correlated with the peak width. Large $|q|$ values yield a nearly symmetric lineshape, while smaller values produce pronounced asymmetry. q is proportional to the ratio of the Raman tensors for phonon and electronic scattering:

$$\Gamma q^2 \propto \left| \frac{R_{phonon}}{R_{electronic}} \right|^2 \tag{S4}$$

Figure S5 shows the excited-state and pre-excitation Raman spectra alongside their fit to the Fano lineshape. The fit captures the data well; a slight peak asymmetry is visible when comparing the intensities of both peak tails. The peak asymmetry is increasing with temperature, as expected from the increasing fraction of free charge carriers above 150 K due to thermal energy larger than the exciton binding energy[9].

The Fano fit parameters of the excited-state spectrum are extracted for each temperature, and plotted in figure S6. The temperature trends of the resonance frequency ω_0 and the linewidth Γ are physically reasonable - the phonon resonance red-shifts and linewidth broadens with increasing temperature. On the contrary, the temperature trends of the Fano asymmetry parameter q^{-1} and the intensity prefactor I_0 are not. The Fano asymmetry factor shows a non-monotonic trend, whereas the raw data, as well as previous reports on its temperature trend in equilibrium Raman scattering, showed a monotonic, increasing trend[10].

This is a confirmation that in transient photodoping, the Fano model is not physical when comparing different lattice temperatures, as it does not incorporate temperature effects[11,12].

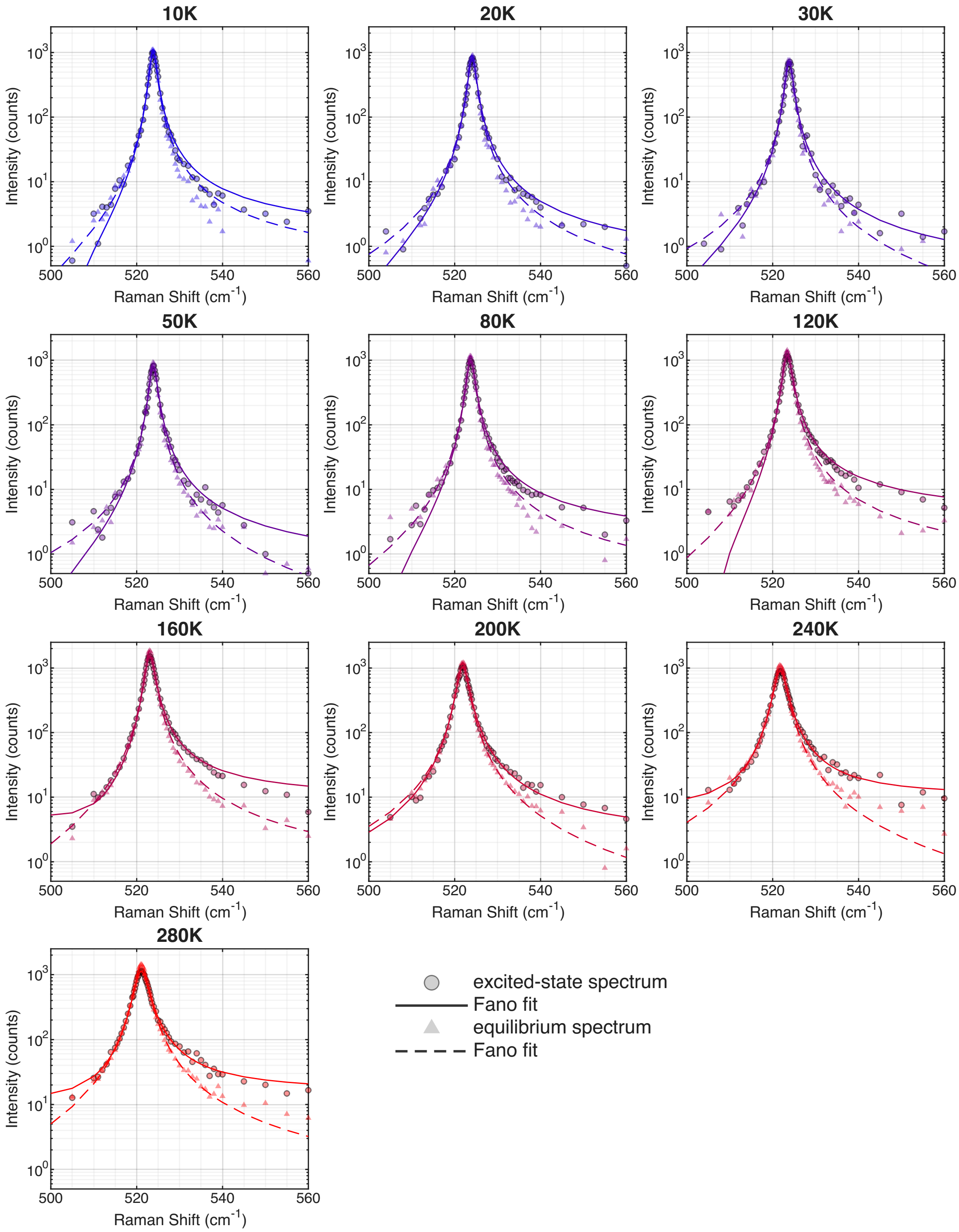

FIG. S5: Temperature-dependent Fano lineshape fit results for the excited-state (circles) and pre-excitation (triangles) spectra show a good fit.

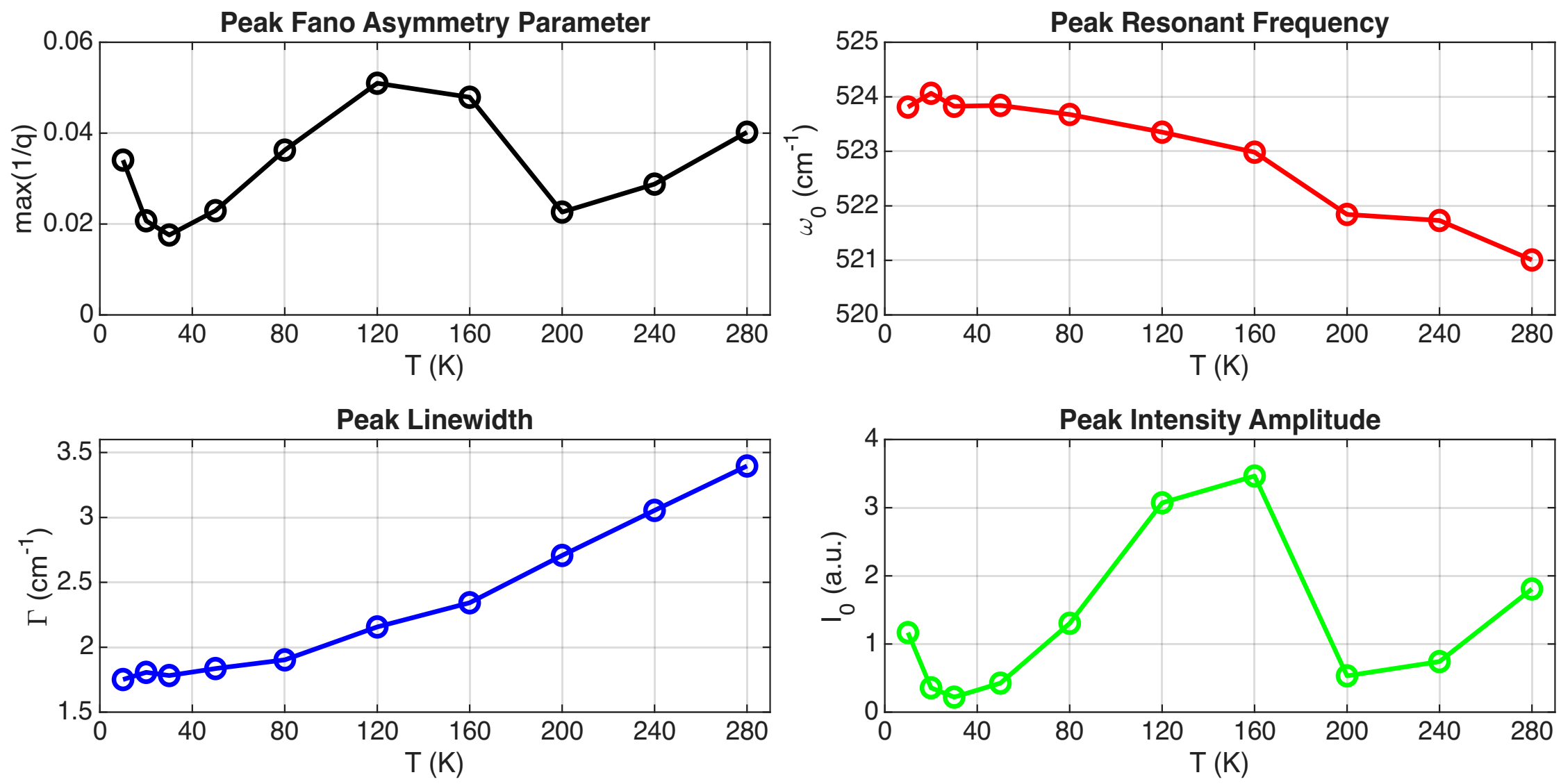

FIG. S6: Temperature-dependent Fano fit parameters extracted at the excited-state spectrum. The q^{-1} and I_0 temperature trends reveal physical anomalies.

2. Coupled-modes model analysis

The coupled-modes (CM) model, based on the single-particle Green's function $G(\Omega)$[13], is employed to describe the interaction between vibrational modes in materials. It is beneficial for systems where vibrational states are coupled to each other. The model captures the coupling's impact on spectral features, including frequency shifts, linewidth changes, and the emergence of lineshape asymmetries.

We vary the temperature of the silicon wafer and follow the variation of the CM fit parameters. Figures S7 – S9 show the temperature-dependent transients of each CM fit parameter, with a bi-exponential decay fit for its signal decay time. We note that only for the imaginary part of the coupling, $\gamma(t)$, both low and high temperatures fitted better to a single-exponential decaying function rather than a bi-exponential; therefore, the dominant exponent was chosen at the intermediate temperatures to plot the temperature trend. As the imaginary part of the coupling term is related to the dissipation of the energy, it may have to do with the change in the dissipative channels at higher temperatures. Notably, all fit parameters are decaying faster upon cooling, where τ_1 values range between 0.3-1.5 ns (R^2), 0.6-7.4 ns (δ), and 0.6-5.0 ns (γ). The faster decay of the photoexcited effect is likely related to a change in the dominant quasiparticle dynamics associated with the formation of excitonic droplets in equivalent temperatures[14–16].

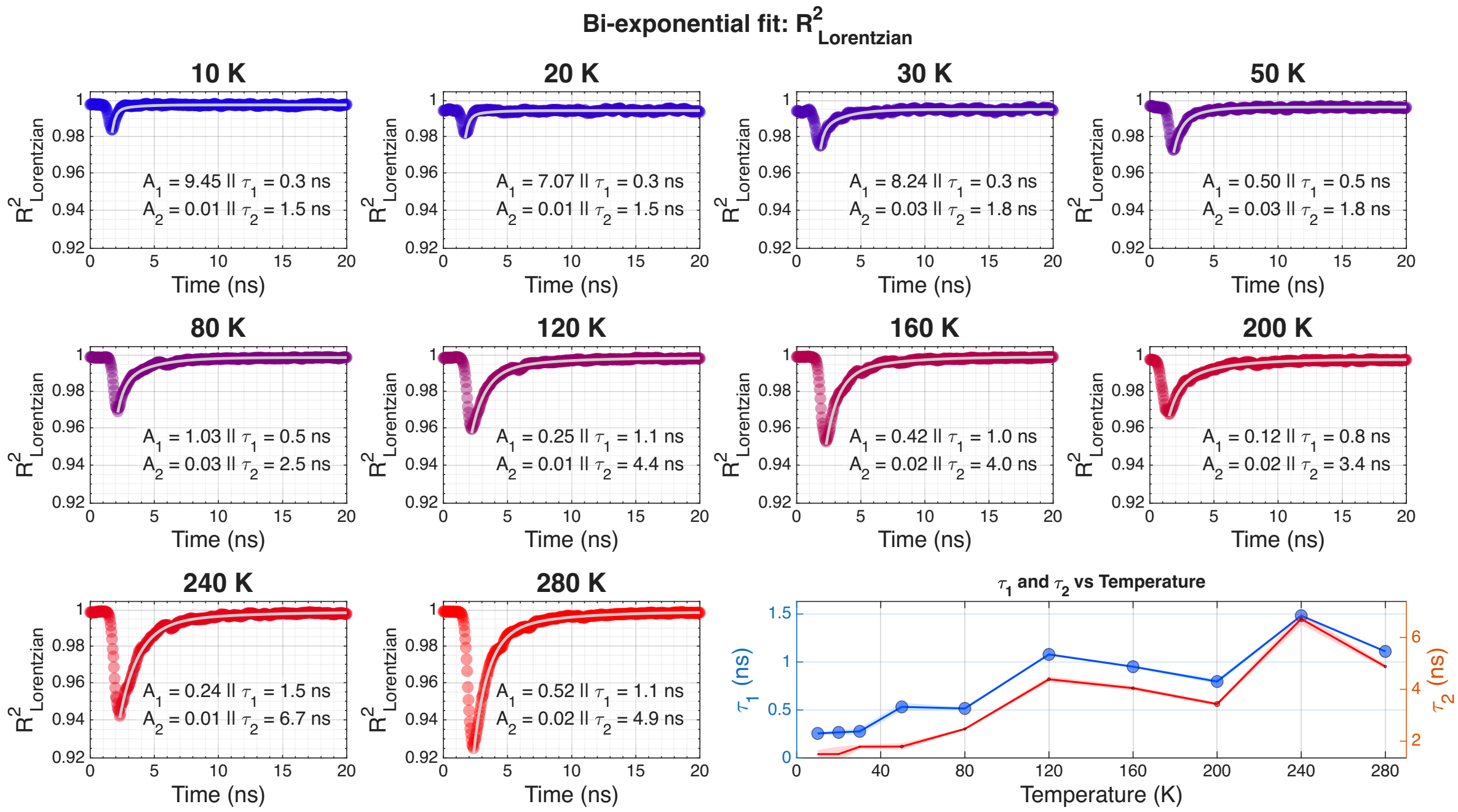

FIG. S7: Temperature- and time-dependent R^2 parameter of the Lorentzian lineshape fit tracks the temporal deviation of the spectrum from the pre-excitation, Lorentzian lineshape. This quantity follows the population of excited charge carriers. All decaying curves were fitted to a bi-exponential decaying function. The bottom-right panel summarizes the time constants between 10–280 K for both τ_1 and τ_2.

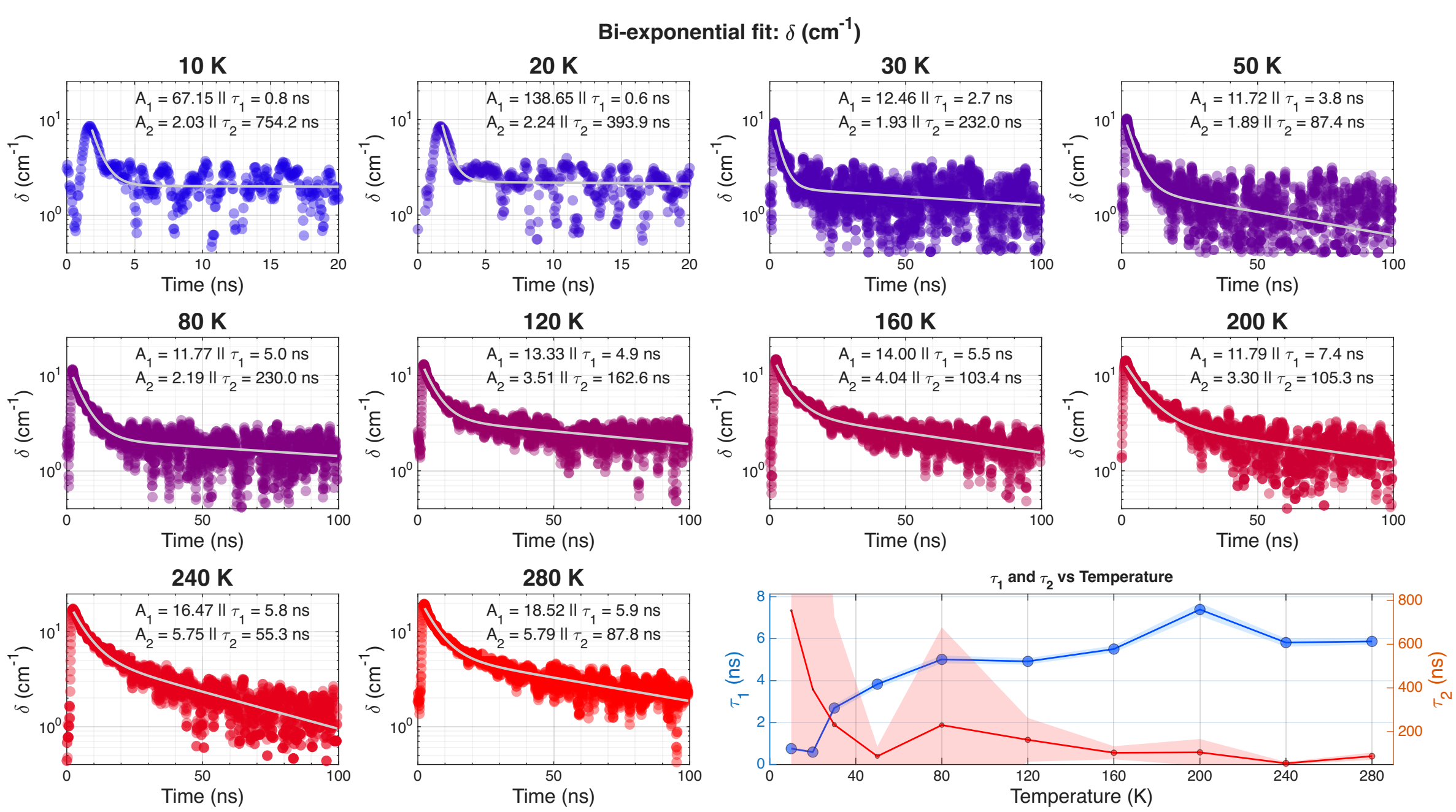

FIG. S8: Temperature- and time-dependent evolution of the coupled-modes fit parameter $\delta(t)$, the real part of the coupling term. All decaying curves were fitted to a bi-exponential decaying function. The bottom-right panel summarizes the time constants between 10–280 K for both τ_1 and τ_2.

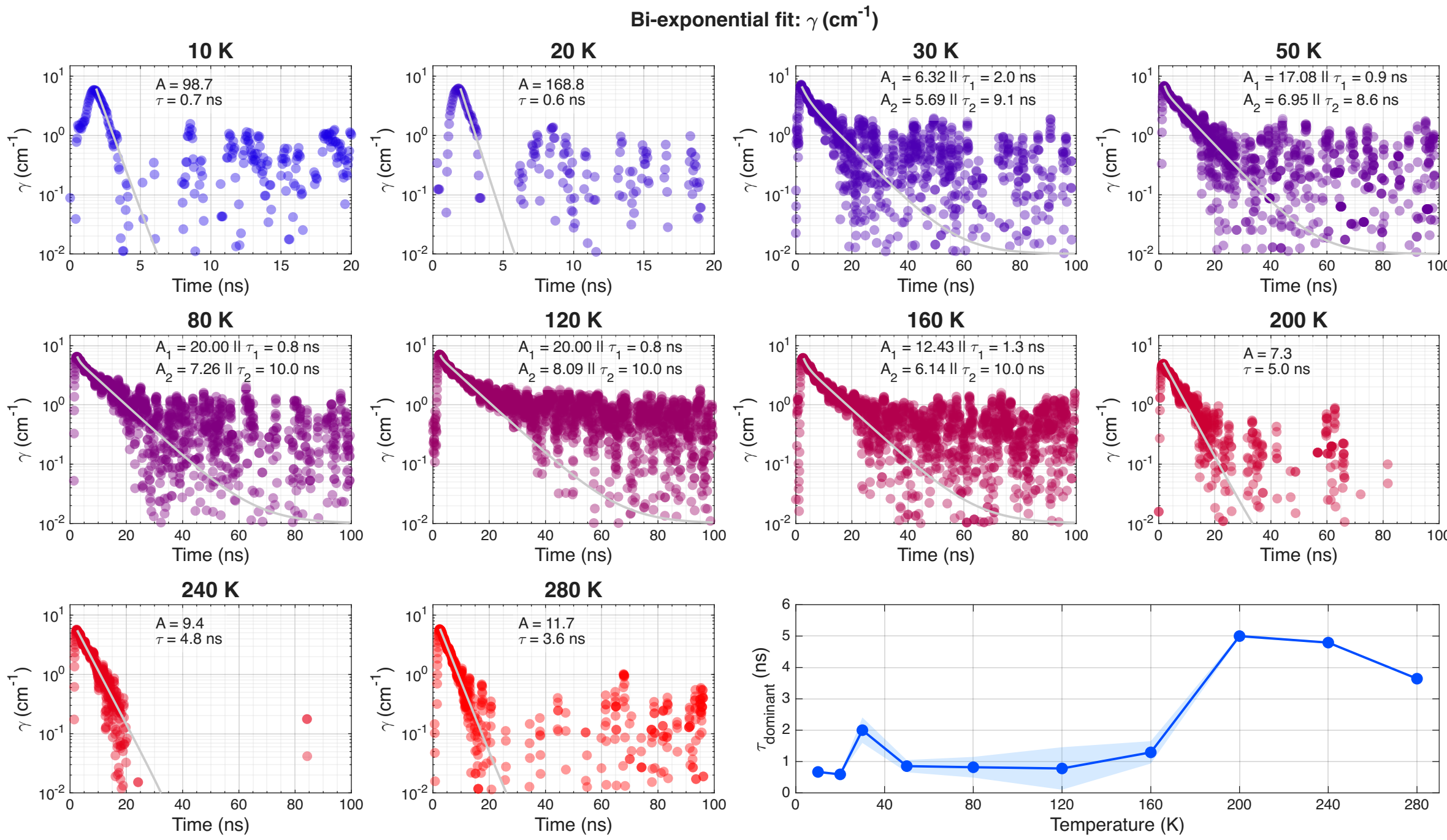

FIG. S9: Temperature- and time-dependent evolution of the coupled-modes fit parameter $\gamma(t)$, the imaginary part of the coupling term. The decaying curves were fitted to either a single- or bi-exponential decaying function, according to the best fit, and the single or dominant exponent value in the bi-exp. curves were extracted to summarize the time constants between 10–280 K.

[1] M. Asher, D. Angerer, R. Korobko, Y. Diskin-Posner, D. A. Egger, and O. Yaffe, Anharmonic lattice vibrations in small-molecule organic semiconductors, Advanced Materials **32**, 1908028 (2020).

[2] M. Menahem, Z. Dai, S. Aharon, R. Sharma, M. Asher, Y. Diskin-Posner, R. Korobko, A. M. Rappe, and O. Yaffe, Strongly anharmonic octahedral tilting in two-dimensional hybrid halide perovskites, ACS Nano **15**, 10153 (2021).

[3] HORIBA Scientific, Diffraction grating calculator (2026), accessed: 2026-07-30. horiba.com/int/scientific/products/custom-spectroscopy-solutions/spectrometers-and-monochromators/grating-calculator/.

[4] S. N. Mohammad, A. V. Bemis, R. L. Carter, and R. B. Renbeck, Temperature, electric field, and doping dependent mobilities of electrons and holes in semiconductors, Solid-state electronics **36**, 1677 (1993).

[5] M. A. Green and M. J. Keevers, Optical properties of intrinsic silicon at 300 K, Progress in Photovoltaics: Research and Applications **3**, 189 (1995).

[6] U. Fano, Effects of configuration interaction on intensities and phase shifts, Physical Review **124**, 1866 (1961).

[7] B. G. Burke, J. Chan, K. A. Williams, Z. Wu, A. A. Puretzky, and D. B. Geohegan, Raman study of Fano interference in p-type doped silicon, Journal of Raman Spectroscopy **41**, 1759 (2010).

[8] F. Cerdeira, T. A. Fjeldly, and M. Cardona, Interaction between electronic and vibronic Raman scattering in heavily doped silicon, Solid State Communications **13**, 325 (1973).

[9] M. A. Green, Improved value for the silicon free exciton binding energy, Aip Advances **3**, 112104 (2013).

[10] V. Magidson and R. Beserman, Fano-type interference in the Raman spectrum of photoexcited Si, Physical Review B - Condensed Matter and Materials Physics **66**, 1 (2002).

[11] M. Balkanski, K. P. Jain, R. Beserman, and M. Jouanne, Theory of interference distortion of Raman scattering line shapes in semiconductors, Physical Review B **12**, 4328 (1975).

[12] F. Cerdeira, T. A. Fjeldly, and M. Cardona, Effect of free carriers on zone-center vibrational modes in heavily doped p-type Si. II. optical modes, Physical Review B **8**, 4734 (1973).

[13] P. C. K. Kwok, Green's function method in lattice dynamics, in *Solid State Physics*, Vol. 20 (Elsevier, 1968) pp. 213–303.

[14] A. Forchel, B. Laurich, J. Wagner, W. Schmid, and T. L. Reinecke, Systematics of electron-hole liquid condensation from studies of silicon with varying uniaxial stress, Physical Review B **25**, 2730 (1982).

[15] S. Revuelta and E. Cánovas, Exciton formation dynamics at the SiO_2/Si interface, Communications Materials **4**, 1 (2023).

[16] I. Pelant and J. Valenta, Luminescence of excitons, in *Luminescence Spectroscopy of Semiconductors* (Oxford University Press, Oxford, UK, 2012) pp. 161–204.
